# Using the ‘Leapfrog’ Design as a Simple Form of Adaptive Platform Trial to Develop, Test, and Implement Treatment Personalization Methods in Routine Practice

**DOI:** 10.1007/s10488-023-01340-4

**Published:** 2024-02-05

**Authors:** Simon E. Blackwell

**Affiliations:** Department of Clinical Psychology and Experimental Psychopathology, Georg-Elias-Mueller-Institute of Psychology, University of Göttingen, Kurze-Geismar-Str.1, 37073 Göttingen, Germany

**Keywords:** Treatment personalization, Adaptive platform trial, Leapfrog trial, Bayes factor, Sequential analysis

## Abstract

The route for the development, evaluation and dissemination of personalized psychological therapies is complex and challenging. In particular, the large sample sizes needed to provide adequately powered trials of newly-developed personalization approaches means that the traditional treatment development route is extremely inefficient. This paper outlines the promise of adaptive platform trials (APT) embedded within routine practice as a method to streamline development and testing of personalized psychological therapies, and close the gap to implementation in real-world settings. It focuses in particular on a recently-developed simplified APT design, the ‘leapfrog’ trial, illustrating via simulation how such a trial may proceed and the advantages it can bring, for example in terms of reduced sample sizes. Finally it discusses models of how such trials could be implemented in routine practice, including potential challenges and caveats, alongside a longer-term perspective on the development of personalized psychological treatments.

Personalization of psychological treatments within routine practice holds promise as a means to optimize treatment outcomes. However, the route to developing personalized treatments, from initial conceptualization to real-world implementation, is complex and full of obstacles that severely limit the chances of success (Deisenhofer et al., 2024). This necessitates revisiting how the treatment development process proceeds for personalization methods and considering new approaches that can increase efficiency and increase the chances of successful real-world implementation.

This paper elaborates on the potential of adaptive platform trials (Angus et al., 2019) in routine practice^1^ as a route to facilitate implementation, evaluation, and optimization of treatment personalization approaches, focusing on a simple version called the ‘leapfrog’ design (Blackwell et al., 2019) as an exemplar. It will start by outlining some of the challenges in evaluating treatment personalization methods, before then introducing APTs and the leapfrog design in particular and describing how they may help overcome some of these difficulties and barriers. Second, it will demonstrate how the principles of such trials could be applied to developing and testing treatment personalization approaches via a hypothetical simulation study. Third, it will move on to discussing how these principles could be integrated into routine practice for efficient and integrated development, testing, and implementation. Fourth, it will discuss some potential caveats and limitations to using such methods for testing treatment personalization, before ending with some concluding remarks.

## Challenges in Testing Treatment Personalization Methods

There are many different ways in which treatment personalization can be conceived and implemented (e.g., Cohen et al., 2021; Lutz et al., 2023), for example allocating patients to different treatments (Schwartz et al., 2021), treatment components (e.g., Fisher et al., 2019; Moggia et al., 2023; Schaeuffele et al., 2021) or therapists (Delgadillo et al., 2020) based on pre-treatment characteristics or individualized assessments, or making changes to a treatment as it progresses based on continuous outcome monitoring (e.g., de Jong et al., 2021; Lutz et al., 2022a, 2022b). However, regardless of the method of personalization used, finding out whether the personalized treatment approach results in better treatment outcomes compared to another, non-personalized, approach requires comparing the two in a randomized controlled trial (e.g., Delgadillo et al., 2018; Lutz et al., 2022a, 2022b). This can pose great challenges for the testing and implementation of treatment personalization methods, and in fact well-powered versions of such trials are rare (Lorenzo-Luaces et al., 2021).

A first of these challenges is that if we are trying to improve upon even a moderately effective treatment, we can only expect fairly small average improvements in symptom outcomes for a personalization method over the non-personalized version (see Nye et al., 2023, for a meta-analysis). This means that an adequately powered trial will require hundreds of participants and, depending on the nature of the treatment and its delivery, a vast amount of time and resources. As an illustration, a ‘standard’ power calculation (80% power, two-tailed, at p < 0.05) to find a small between-group effect size (*d* = 0.2) will return a sample size of *n* = 394 per arm; a simple two-arm trial based on such a calculation would therefore need to randomize 788 participants—and this is before attrition is factored in.

Part of the sample size challenge is also that for any one personalization approach, it may be assumed that there is a limited group of patients for whom the personalization will make a difference; some patients will show a good response and others no response regardless of the treatment offered (e.g., the “spontaneous remitters” vs. “intractable” groups suggested by DeRubeis et al., 2014). The scope for improvement via personalization can be indicated by estimation of heterogeneity of treatment outcomes (e.g., Kaiser & Herzog, 2023; Kaiser et al., 2022). However, in general any advantage of the personalization method will be carried by only a subset of the overall sample (e.g., those whose treatment progress goes “off-track”; Delgadillo et al., 2018). Conceivably one might think that it would be possible to increase power and reduce the sample size by identifying and only including those patients for whom the personalization is predicted to make a difference. However, the process of identifying the patients to be offered personalization ultimately becomes part of the personalization approach itself, and once the package of identifying such patients and offering personalization is compared to TAU the problem of the small overall effect sizes returns.

A second consideration is that, regardless of feasibility, such a large trial remains a massive gamble: Even if we have strong evidence from preliminary work or other clinical services suggesting that the personalization should bring improvements, we cannot assume successful translation from earlier- to later-phase work or across treatment settings. As an example (albeit not from psychological treatment research), the PReDicT trial (Browning et al., 2021) tested whether guiding pharmacological treatment of depression via performance on tests of cognitive biases and symptoms soon after initiation of treatment would lead to improved outcomes versus TAU in a sample of 913 patients with depression. The trial built on an extensive amount of previous pre-clinical and clinical studies that provided what appeared to be a promising basis for such a pragmatic trial; however in the end there were no differences in depression outcomes between those patients whose treatment was guided by the PReDicT algorithm and those receiving TAU. On reading into the details of the trial, one of the major challenges of moving from prior work to a pragmatic real-world test becomes readily apparent: that many decisions about *how exactly* the personalization method will be implemented need to be made. Some of these decisions may be service- or setting-specific. For example, the precise implementation of personalization methods involving matching patients to therapists or treatments will necessarily depend on the therapists available, including their relevant training and expertise. Although decisions about implementation can be guided to some extent by previous research they will also likely involve a degree of both compromise and guesswork (and post-hoc, it will always be possible to identify many ways in which the personalization could have been better implemented). This poses a major risk, as guessing wrong may lead to the personalization approach being abandoned when in fact it was the implementation that was suboptimal. That is, in such a trial we may think that we are testing an *idea* but in fact we are only ever testing one very specific *implementation* of that idea (Blackwell & Woud, 2022); even if the treatment personalization method itself is in principle effective, sub-optimal implementation may lead to a very expensive false-negative null result. After such a null result, planning, setting up, then running a follow-up trial to test either a differently-optimised version of the personalization, or a different personalization method, may then take several years. Further, after a null result it may be even more difficult to acquire funding for a follow-up trial as grant reviewers and funding agencies may argue that the idea has already been tested and found ineffective.

A third consideration is that ideas for treatment personalization will likely evolve faster than the time-course of a large trial (especially a trial involving face-to-face therapy). This leads to a bottleneck in research progress and means that by the time a trial finishes the idea being tested may have already been superseded by further innovations (“translational block”; Blackwell et al., 2019).

Fourth and finally, even if an effective personalization method is developed and shown to be effective in an RCT, the gap to actual implementation in routine practice is huge and will most likely take a long time to cross—if indeed it is ever crossed at all. While this implementation gap is by no means unique to treatment personalization (e.g., Damschroder et al., 2009), there are aspects of treatment personalization that may raise specific problems. For example, some approaches may require specific complex technical or statistical expertise that staff in a routine treatment setting may lack, may place additional burdens on already-busy clinicians, or may be viewed as undermining the clinicians’ expertise and skill in assessing and treating patients (see Deisenhofer et al., 2024, for further discussion).

Overall, these factors meant that attempting to test, implement, and optimize personalization methods via a sequence of standard RCTs will often be hopelessly inefficient. These challenges become further magnified once moderately effective treatment personalization methods have been found and the average gains in efficacy to be expected become even smaller. Optimally we would have pathways for continuous development and testing of treatment personalization methods in real-world implementation.

## The ‘Leapfrog’ Trial as a Route for Treatment Development

A relatively efficient way to evaluate and optimize personalization methods would be to do so in combination with implementation in the context of adaptive platform trials (APT; Angus et al., 2019; Hobbs et al., 2018) embedded within routine care delivery (Blackwell et al., 2019; see also Deisenhofer et al., 2024; Herzog et al., 2022; Liu et al., 2021). Adaptive platform designs provide a means to consolidate what would normally be a long drawn-out treatment development process comprising several RCTs into a single flexible trial infrastructure, and have gained particular prominence in recent years through their use in the COVID-19 pandemic (see e.g., Calfee et al., 2022; Gold et al., 2022). While there are many different ways in which adaptive platform trials can operate, the general principle is that once such a trial has started, treatments can enter and leave the trial (potentially alongside other adaptations to the trial design) while the trial is proceeding, based on ongoing analyses and implementation of pre-set decision rules. However, the complexity of such trials presents a significant barrier to their use within routine psychological treatment development work, and hence a simplified version termed the ‘leapfrog’ design was devised to facilitate their uptake (Blackwell et al., 2019).

A basic leapfrog design runs as follows: One or more intervention arms are compared to a designated control condition or comparator arm (e.g. the current version of the treatment that the researchers wish to improve upon), with participants randomly allocated across the arms. Once an arm (and the comparator arm) have hit a pre-set minimum sample size (*N*_min_), sequential Bayesian analyses are initiated based on calculation of directional sequential Bayes factors (BFs; see e.g., Schönbrodt et al., 2017), comparing that arm to the comparator on a pre-designated primary outcome (e.g. post-treatment scores on an outcome measure or change in symptoms from pre to post-treatment). These Bayes factors quantify the strength of evidence provided by the data gathered so far for one hypothesis (e.g. that the treatment is superior to the comparator arm) versus another (e.g. that the treatment is not superior to the comparator arm). If the BF for an arm hits a pre-designated threshold for failure (i.e. non-superiority), BF_fail_, that intervention arm is dropped from the trial, meaning that no further participants are randomized to receive it. If the BF for an arm hits a pre-designated threshold for success (i.e. superiority), BF_success_, it is ‘promoted’ to become the new comparator arm, with the previous comparator arm dropped from the trial. This means that if there are remaining intervention arms in the trial, they are now compared to this new comparator arm, which thus becomes the new benchmark for interventions to improve upon. If an arm reaches a maximum sample size, *N*_max_, it is dropped from the trial (i.e. no further participants randomized to receive it), providing a time (and resource) limit for testing of each treatment. New treatment arms can be entered into a trial that has already started, and once they reach *N*_min_ they too will be compared to the comparator arm (against those participants in the comparator arm who were contemporaneously randomized).

Once initiated, a leapfrog trial therefore provides a continuous process of treatment development and optimization that can either run for a limited period (e.g. until a pre-defined level of treatment success is reached by one of the arms) or indefinitely. The ‘leapfrog’ name comes from the way in which effective trial arms successively ‘leap’ over the comparator arm to become the new comparator.

Compared to a traditional treatment development process (typically a series of 2- or sometimes 3-arm RCTs running to a fixed sample size), a leapfrog design confers several advantages. First, the use of sequential analyses allows relatively rapid detection of ineffective treatments, substantially reducing the sample sizes needed (see e.g., Blackwell et al., 2019; Schönbrodt et al., 2017) and also reducing the number of patients who receive a less effective treatment. Second, the ability to add arms into an ongoing trial can accelerate the treatment development process by allowing new research findings to be incorporated into an ongoing trial via a new arm (reducing the “translational block”; Blackwell et al., 2019). Importantly, the design of new treatment arms can be informed not only by external research results, but also via data emerging from the trial itself. From a treatment personalization perspective, as data accumulates in the trial this can be analysed via data-intensive methods often used within personalization research such as machine-learning (e.g., Giesemann et al., 2023) to try to detect moderators, test counterfactual allocations of participants to treatments, or investigate potential mechanisms. The results of such analyses can then form the basis of hypotheses about how a treatment can be improved, which can be tested directly in the ongoing trial via introduction of a new trial arm (“real-time data-driven cumulative treatment optimization”; Blackwell et al., 2023). Third, the potential for continuous optimization (via promotion of arms to become the new comparator arm) means that what would normally be a treatment development process comprising a series of separate large trials is consolidated into one trial infrastructure (eliminating the “inter-trial lag”; Blackwell et al., 2019). These features of the design make starting a leapfrog trial much less of a gamble than for a standard fixed-N RCT. Not only does the lower sample size requirement reduce the resources invested, but this in turn enables testing more than one ‘implementation’ of the ‘idea’ if desired. Further, the ability to add new arms into an ongoing trial means that trial initiation is not an irreversible commitment as later-occurring ideas can still be incorporated.

In terms of the analysis parameters themselves (the BF and sample size boundaries), these can be chosen based on a number of considerations, such as the effect sizes of interest, feasibility issues, and the researcher’s preference for certainty vs. speed. The researcher also needs to choose a primary outcome (i.e. the outcome measure that will be used for comparing the trial arms) and the preferred method for calculating the BFs (including the analysis prior). As outlined in previous papers (Blackwell et al., 2019, 2023), the researcher can use simulations to determine a set of analysis parameters that will provide the statistical power to find the effect size of interest and the desired false-positive rate. For example, Blackwell et al. (2019) provide an example set of parameters for two hypothetical trials designed to provide 80% power to find effect sizes of 0.4 or 0.3, with a false-positive rate of < 0.05 (i.e. the conventional power and false-positive rate often used in psychological treatment research). Alternatively, from a fully Bayesian perspective a researcher could start by deciding on the BF boundaries they consider to give a suitable level of evidence (e.g. a BF > 10 for strong evidence), then use the simulations to determine the sample size limits and thus the feasibility and resource implications of the trial.

## Applying the Principles of the Leapfrog Design to Testing Personalized Psychological Therapies

This next section will illustrate how a leapfrog design could be applied to development and testing of personalized psychological therapies. This example is designed to illustrate the principles and potential advantages of the leapfrog design, and as such is deliberately kept as simple as possible, following the same method as outlined previously (Blackwell et al., 2019). The hope is that this keeps the ideas accessible to the widest possible readership, including people unfamiliar with Bayesian analyses or simulation; this also avoids getting side-tracked by considerations of specific complex trial implementations or statistical methods, which will tend to be idiosyncratic to a specific trial and are not directly relevant to the aims of this paper.

### Setting the Scene

Imagine this situation: You are running a psychological therapies service (or network of services), and wish to test out how a new personalized therapy approach compares to the therapy as currently delivered in your service. There are of course many forms the personalization could take. For example, it could be a method for allocating (or recommending) each patient to the different treatment options (or therapists) available in your service; it could be an outcome monitoring-based personalization approach in which patient symptom trajectories are used to inform changes to the therapy procedures; or it could be a new modularized transdiagnostic therapy with data-driven module selection and sequencing. However, the exact method of personalization or what is meant by it does not matter for the purpose of the example: you have a way in which therapy is currently delivered and wish to improve upon it. Because your original therapy is moderately effective, you think that realistically you can only expect small (Cohen’s *d* = 0.2) average effect size improvements upon it (likely a realistic effect size estimate for personalisation; Nye et al., 2023). As outlined previously, if you were to consider running a standard fixed-N trial with conventional power and false-positive rate (i.e. 80% power to find a between-groups difference or *d* = 0.2, two-tailed, at *α* < 0.05), your power calculation would tell you that you needed a total of 788 participants (*n* = 394 per arm).^2^ If at the end of this trial you found that your personalized treatment was in fact no more effective than the treatment as currently provided, you might run another trial to test another version of this treatment. Or if this was effective you might then run another trial to see if you can improve upon this further. With this standard approach, if you ended up testing 3 different personalization approaches one after the other, this would end up meaning 2,364 participants (and many years of research); once you have tested 5 personalization approaches you would be up to 3,940 participants in total.

### Taking a Leapfrog Approach

How would this process run as a leapfrog trial? Here we will imagine that we start by testing out three versions of the personalized therapy against the ‘standard’ therapy,^3^ and when any of these drop out, we can introduce further versions. For the purposes of illustration we will treat the actual analysis as something of a black box, and use the BFDA package by Schönbrodt and Stefan (2019) for the simulations. The BFDA package provides a simple method to simulate sequential BF-based analyses and explore their characteristics for certain analysis methods. We will use the method applied to a t-test, allowing simulation of Cohen’s d between group-effect sizes (e.g. between post-treatment scores on a symptom measure, or however the effect size of interest is conceptualized for the planned analysis).

#### Determining Analysis Parameters

Here we take the approach outlined by Blackwell et al. (2019) of finding a set of analysis parameters that lead to an approximation of standard power (80%), and pairwise false-positive rate of < 5%, to find a between-group effect size of *d* = 0.2. Practically speaking, this can be done by starting with some approximate parameters (e.g. those for *d* = 0.3 provided by Blackwell et al., 2019), and adjusting these to find those that have the desired characteristics at *d* = 0.2 (the target effect size), and *d* = 0 (for checking false positives). The effects of these parameters over a range of other effect sizes can be found. By this process, we end up with a set of parameters that give the desired power and false-positive rates: *N*_*min*_ = 150*, N*_*max*_ = 450*, BF*_*fail*_ = 1/5 and *BF*_*success*_ = 3 (in this case using an analysis prior with rscale = 0.5).^4^

#### Examining the Predicted Effects of the Parameters on Trial Performance

Table 1 below shows the consequences of these analysis parameters on a pairwise basis, which indicates that, for example, if the personalized treatment really is no better than the standard one (*d* = 0), BF_fail_ will be reached approximately 50% of the time at N_min_. If the true effect size really is exactly as expected (*d* = 0.2), then 50% of the time you would reach BF_success_ by about 200 participants per arm. Note that if the personalization is better than expected (e.g. d = 0.3), it is also highly likely that BF_success_ would be reached by an early time point, and the probability of reaching BF_fail_ is extremely small. Conversely, if the personalized treatment is in fact *worse* than the standard treatment, BF_fail_ is reached very quickly, and the chances of erroneously hitting BF_success_ are negligible.

**Table 1:** Illustration of probabilities (as percentages) of different outcomes for an example set of trial parameters

Trial parameters*: N*_*min*_ = 150*, N*_*max*_ = 450*, BF*_*fail*_ = 1/5 *and BF*_*success*_ = 3											
‘True’ effect size (Cohen’s *d*)		Probability of reaching ‘discontinuation’ threshold at each participant number (per group)					Probability of reaching ‘replacement’ threshold at each participant number (per group)				
	*N*	150	200	250	350	450	150	200	250	350	450
− 0.2		96.5	99.1	99.6	99.9	100.0	*0.0*	*0.0*	*0.0*	*0.0*	*0.0*
− 0.1		85.4	93.5	96.4	98.6	99.4	*0.0*	*0.2*	*0.3*	*0.3*	*0.3*
0		56.6	70.5	77.5	84.3	88.6	*2.0*	*3.1*	*3.5*	*4.4*	*4.9*
0.1		23.4	34.1	39.7	45.0	47.2	9.8	17.2	22.0	29.8	**35.0**
0.2		5.9	8.9	10.2	10.7	11.1	33.3	50.8	60.1	72.7	**80.8**
0.3		0.9	1.3	1.3	1.4	1.4	67.3	83.6	91.4	96.7	**97.9**
0.4		0.0	0.0	0.0	0.0	0.0	89.9	97.3	99.2	99.9	**100.0**
0.5		0.0	0.0	0.0	0.0	0.0	98.3	99.9	100.0	100.0	**100.0**

The italics values indicate the false-positive rate (i.e. concluding d > 0 when d ≤ 0) as recruitment and the sequential analyses proceed, with the sample size increasing from the specified N_min_ to N_max_. The bold values indicates power to detect d > 0 by N_max_ at for different levels of ‘true’ effect size. Negative values of d indicate superiority of the comparator arm

**Fig. 1:**
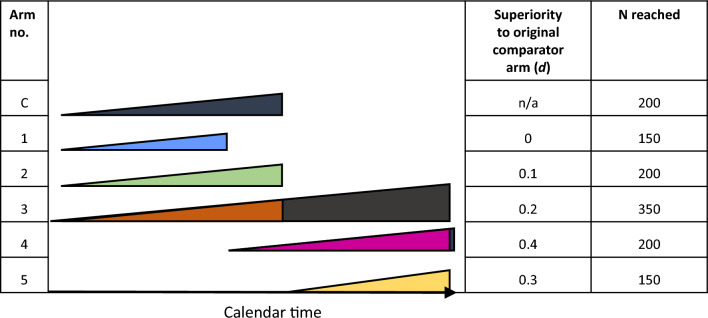
Illustration of a hypothetical trial using the leapfrog design. Each triangle depicts a study arm, with the height of the triangle indicating the number of participants recruited. The comparator arm is indicated by the dark grey shading of the triangle, and the initial comparator arm is designated “C”

Figure 1 shows how such a trial might operate on average (i.e. the outcomes shown approximate to what we would expect to happen ~ 50% of the time for each comparison). Here, in the initial phase, 3 versions of personalized therapy (arms 1, 2, 3) are compared against the standard therapy (C). After 150 participants (per arm), arm 1 reaches BF_fail_ and is dropped, with another arm (4) being introduced. At 200 participants (per arm), arm 3 hits BF_success_ and leapfrogs the ‘standard’ arm to become the new comparator arm, at which point arm 2 hits BF_fail_ and is dropped. One further attempt to improve upon arm 3 (arm 5) is now introduced. After arm 4 has 200 participants, it hits BF_success_ and leapfrogs arm 3 to become the new comparator arm, at which point arm 5 hits BF_fail_ and is dropped; arm 4 is now the ‘standard’ treatment against which further attempts to improve treatment outcomes could be compared.

This example trial used a total of 1,250 participants to test 5 personalization methods. What sample size would have been used with a standard fixed-N trial with conventional a conventional power calculation to find d = 0.2 to replicate the same set of results? In the most efficient approximation (i.e. an initial 4-arm trial followed by a second 3-arm trial), such a trial would require 2,758 participants, that is, around double the number; if following the standard treatment development process (i.e. a series of 2-arm trials to test the 5 personalization methods), it would require 3,940, around 3 times as many.

#### Further Points Illustrated by the Example Trial

While this example trial is a somewhat simplified illustration, it highlights a few interesting points. As illustrated in Table 1, the analysis parameters chosen meet the criteria of ‘standard’ power and alpha, but from a standard Bayesian perspective a BF threshold of 3 might seem a very low level of evidence for making decisions; it is, but this reflects the fact that the standard frequentist p < 0.05 threshold often does not provide very strong evidence for the alternative hypothesis compared to the null. If strong evidence was wanted from a Bayesian perspective (e.g. BF > 10), the trial of course could be set up with more stringent boundaries; however, the expected sample sizes would increase accordingly. In fact, there is no necessity to decide upon an 80% power and < 5% false-positive rate—the exact error rates that a researcher will tolerate will likely depend on a whole range of factors (e.g. if the cost and resource-implications of implementing a new version of the therapy are low, as may be the case for some automated computerized interventions, a higher false-positive rate might tolerated). A second point to note is that with a minimum sample size of 150 per arm it is not ‘cheap’ to test interventions or add in new arms. It is possible to have lower N_min_ values if your priority is being able to reject ineffective interventions quickly; however, in order to achieve reasonable error rates while retaining power to detect small effect sizes, this might mean needing much more stringent BF boundaries, and in turn a much higher N_max_ to achieve reasonable power. Hence, there is no ‘right’ or ‘best’ set of parameters, but rather the researcher must adapt these according to their priorities (e.g. speed vs. certainty). Note that the potential heterogeneity between different trials in the thresholds chosen means that interpretation of the results of any individual leapfrog trial requires paying close attention to the parameters used for the analyses and the estimated error rates.

#### Using more Complex Analytical Approaches

Of course, in a real-life trial it is unlikely that the actual analysis will involve a t-test between two treatment arms. Rather, more likely is some kind of multi-level model that allows for incorporation of participants with missing data, stratification variables (including site if it is a multi-site trial), nesting of participants within sites (and potentially within therapists) and so on (e.g., Delgadillo et al., 2018). If what is being tested is a treatment allocation method (e.g. allocating people to CBT vs. another psychological therapy based on some kind of algorithm), the unit of comparison (i.e. the trial arms) would not be the therapy itself (e.g. CBT vs. other). Rather, one arm would comprise people being allocated to the two treatments via the new method, and the control arm would comprise people being allocated by whatever the standard method would be (or randomly), and the outcomes of people in one allocation method arm would be compared to the outcomes of those in the other allocation method arm. In these more ‘real-world’ circumstances how would a researcher go about planning and analyzing the trial?

This process can be broken down into several steps. First, unless the researcher is a Bayesian analysis expert, they can start off by ignoring the fact that they will be conducting sequential Bayesian analyses and decide upon what they think would be the optimal analysis method if this was a standard fixed-N trial (e.g. multi-level model controlling for stratification variables and with the desired random effects). The next question would be to ask what the most appropriate Bayes factor (or other Bayesian index if preferred) would be to extract from this for the purpose of the sequential analyses. Extracting the Bayes factor could be achieved via fitting fully-Bayesian models, or via calculating approximate Bayes factors from model parameters (as illustrated by the simulations and protocol accompanying Blackwell et al., 2023, in relation to mixed models); while a fully-Bayesian model will have some advantages (e.g. greater ease of extracting further model indices such as posterior distributions), there are also disadvantages (e.g. computational complexity and time), and an approximate Bayes factor may perform adequately. In fact, if there are uncertainties about what the best analytic approach may be, the different possibilities can be compared via the simulations. Having chosen the analysis method for extracting a Bayes factor, the next step would then be to apply this chosen method sequentially (i.e. repeatedly as the sample size accumulates) to simulated data, simulating over a range of effect sizes and other possible assumptions (e.g. patterns of missing data, correlational patterns within datasets). A set of analysis parameter can then be chosen that achieve the desired power, false-positive rate, and sample size constraints. Once these parameters have been determined at a pairwise level, their effects can then be simulated over a trial comprising several arms with a range of effect sizes, to see how an entire multi-arm trial might perform over time. Having pre-registered this analysis plan as part of the trial registration process, the trial can then begin: Once the minimum sample sizes are reached, the analyses are carried out as used in the simulations, and action taken as appropriate when the BF or sample size boundaries are reached.

A simple demonstration of this can be seen in the protocol and accompanying scripts for the study by Blackwell et al. (2023), in which three analysis approaches (t-test on change scores, mixed models, constrained longitudinal data analysis) were compared at different levels of missing data, using approximate BF calculations. Interestingly, in the absence of missing data the more complex approaches performed similarly to the t-test, indicating that simple approaches (e.g. t-test using the BFDA package as used for the demonstration here) can provide a useful starting point for initial planning and to get a ‘feel’ for the methods (and can also be adapted to incorporate informed analysis priors; Stefan et al., 2019). In fact the complexity of the analyses for more sophisticated trials (e.g. multi-site with more complex models) is not a function of the leapfrog or broader APT design itself, but something that would have to be worked out for even a basic trial, and hence if the study was going to be done at all, it is worth investigating how it could be transformed into a leapfrog trial.^5^

## Integration with Routine Practice

This next section outlines how the principles of the leapfrog design could be combined with treatment delivery in routine practice and thus provide a sustainable pipeline for development, implementation, and evaluation of treatment personalization.

### Rationale for Integration in Routine Practice

There are a number of reasons why adaptive platform designs such as the leapfrog design may be particularly suitable for ongoing continuous treatment development in routine practice (Blackwell et al., 2019, 2023; Deisenhofer et al., 2024).

First, integration of treatment development and treatment delivery in routine practice via the leapfrog design (or APTs more broadly) provides a chance to streamline resources and to reduce the time delays between developing a new treatment, demonstrating efficacy, and rolling it out in real-world settings. Once the basic infrastructure is established, the trial can run continuously without the disruptions that might come from starting/stopping individual trials, as the main activities are continuing analyses over time and making changes to the treatment arms included. Meanwhile, the accumulating data can be analysed to inform hypotheses about further methods for optimizing the approach under investigation. When a new treatment version reaches the superiority threshold it can become the new ‘treatment as usual’, and can be rolled out as the standard of care to be improved upon; hence such a trial provides a mechanism for a continuous process of treatment development that should see cumulative improvements in routine treatment delivery over time. While in many cases there will of course be resource and practical implications for including a new treatment arm in a leapfrog trial (discussed later on), these will still be much less than for starting up an entire new trial—which would be the only option under standard research approaches.

Second, integration of the leapfrog design into routine practice also provides a means to overcome the problem of potential lack of generalization of research findings about the relative efficacy of different approaches over time and across settings: Given that a between-group effect size is essentially the ratio of the outcome variance attributable to the different interventions and the outcome variance attributable to everything else (which will include socio-cultural, political and historical factors), the effect sizes from any given study are necessarily situated within a very specific context. From this perspective it may be useful to move away from the current reliance on ‘historical’ evidence (i.e. via the accumulation and synthesis of more and more data stretching across decades to reach meta-analytic effect size estimates informed by ever-greater sample sizes) to ‘live’ evidence that is constantly addressing the question: how can we improve upon what we are currently offering (Blackwell & Heidenreich, 2021). In fact, use of (relatively) ‘live’ evidence is already implemented in some services, for example the UK’s NHS Talking Therapies (formerly Improving Access to Psychological Therapies), where standardized data collection has been used to inform improvements in service delivery (e.g., Clark et al., 2018), but the embedding of novel trial designs within such services can offer many further opportunities (Herzog et al., 2022). In particular, the evidence informing practice could be not only current (or at least, relatively recent) but also locally-tailored, rather than risk being determined by largely historical evidence collected in what can sometimes be very different contexts.

Third, the features of a leapfrog trial should make it particularly attractive for both patients and services. In the context of a service in which outcomes and therapy delivery are already monitored, the basic additional requirement for integration of the leapfrog design into routine treatment delivery is that patients consent to be randomized. However, given that randomization is to either i) the routine treatment that the patient would be receiving anyway if they do not consent to be randomized or ii) one or more newer attempts to improve upon this, this seems a relatively low barrier. Additionally, as less effective arms tend to be dropped from a trial and more effective arms tend to survive for longer, patients end up having a higher likelihood of being randomized into more effective, rather than less effective, treatments (Blackwell et al., 2023). These features are also particularly advantageous from an ethical perspective. From a service point of view, if an idea comes up for improving the treatment delivery, rather than simply implementing it without any randomized evaluation (or only pre/post cohort testing, which is problematic due to history effects and the implications if the ‘improvement’ actually makes things worse), the innovation can be implemented alongside the existing treatment and if it does appear superior it can take over; if patients are coming through the service anyway and may be offered one or more treatment options there is little to lose (and a lot to gain) from randomizing them. The flexibility offered by the design also potentially offers the chance for an ongoing trial to be reactive to changes in legislation regarding treatment provision or training. To take an extreme example, if changes in infrastructure or legislation meant that none of the current arms were still applicable and a whole new set of trial arms were needed, these changes could be implemented within the existing framework of the trial rather than having to start up a new trial from scratch. Finally, the flexibility and potentially open-ended nature of the leapfrog trial may make it a more acceptable way to increase the extent to which the treatment being offered in a particular service is evidence-based. As the initial comparator arm/control condition would generally be whatever is currently being offered in the service, starting such a trial does not make the assumption that what clinicians are currently doing is wrong and needs to be replaced, but rather asks the question of whether there are ways in which the current offerings can be improved upon. The current ‘standard practice’ at the service therefore evolves over time, guided by the data that emerges, rather than simply being imposed from without.

### Models for Implementation of Adaptive Platform Trials across Routine Practice

**Fig. 2:**
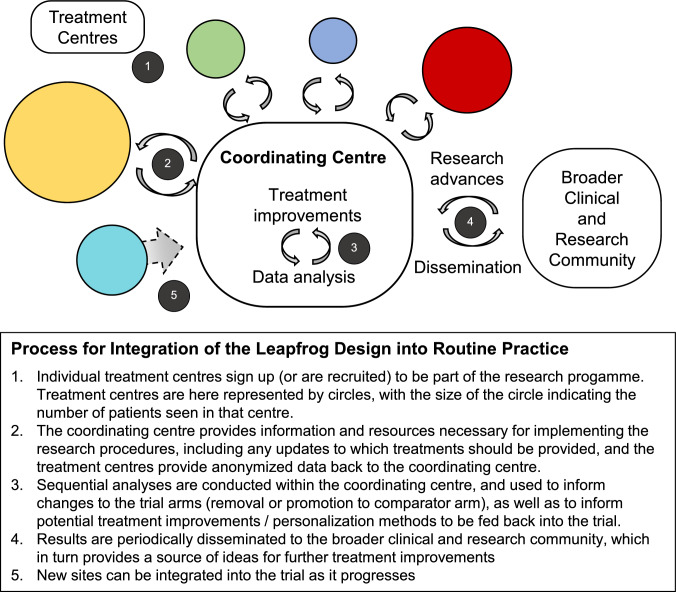
Schematic overview of integration of the leapfrog design into routine practice across multiple sites

Can implementation of treatment personalization approaches within routine practice via adaptive platform designs be reconciled with the fact that for many patients, the only opportunity to receive treatment may be in relatively small local treatment centres that will require an unviably huge amount of time to achieve the sample sizes required? If we wish all patients to have the opportunity to benefit from personalized treatment approaches and contribute to their further development, then it is useful to consider some models that could allow this to happen. A schematic overview is presented in Fig. 2.

#### Digital Mental Health Interventions from Central Providers

When the treatment being provided is web or app-based, there may be one central provider (e.g. a commercial company) providing the software for the interventions (e.g. internet-delivered CBT), as well as outcome measurement, to many services or treatment centres. It is likely that such software or the interventions are tweaked over time, including attempts to personalize them; the leapfrog design provides a means to continuously evaluate such tweaks and adaptations without disturbing the ongoing service provision: As soon as enough evidence is reached to be sufficiently confident that a ‘new’ version is better than the current version on the chosen outcome (e.g. a clinical outcome, or something else such as number of ‘modules’ completed or patient satisfaction), this new version can become the new ‘standard treatment’. In such circumstances there is probably little in the way of cost implications for adding in or switching to a new version, and this could proceed seamlessly without disrupting any aspect of treatment delivery. Optimally, methods found to improve the interventions would also be disseminated not only via research publications, but also more widely via e.g., provision of training opportunities, resources (e.g. protocols, software systems), and feeding back to policy-makers, so that they could bring benefits beyond those patients who happen to receive the intervention from this particular service provider.

#### Distributed Services with Centralized Oversight

In other settings there may be distributed, localized, provision of services but some form of centralized oversight and mandating of provision and outcome measurement (e.g. the UK’s NHS Talking Therapies). With such a service delivery model individual sites could be selected (or sign up) to be part of the ongoing leapfrog trial and thus offer their patients the option of consenting to randomization. While there would be logistical considerations in rolling out a new version of a therapy across participating sites, this would be the case for any attempt to improve the treatments offered and is not a function of the leapfrog design itself. In fact, a leapfrog design may provide certain advantages over other complex trial designs that could be used to evaluate improvements in treatment delivery across services (e.g. stepped wedge cluster randomized trials; Hemming et al., 2018). For example, randomization at the patient-level (i.e. within individual sites) facilitates investigation of and accounting for the fact that there may be differences between sites in how beneficial any specific personalization method may be. Further, this increases flexibility by allowing additional sites to join the trial (or leave it) in a non-random way without compromising the random allocation itself. Finally, randomization at the patient level also facilitates adding in of new arms as this can also happen in a step-wise manner based on feasibility issues (which are also likely to be non-random).

#### Decentralized Networks of Independent Treatment Providers

The final model discussed here involves independent treatment services signing up to be part of a network with some level of harmonization of data collection and potentially other procedures. An example is provided by the German ‘KODAP’ initiative, a cooperation between university outpatient psychological therapy clinics (Margraf et al., 2021). Within such a model, a research centre or network might offer a new personalization approach (e.g. an algorithm or feedback approach, or method for treatment selection), and clinics could sign up to take advantage of this if they agree to conditions of data harmonization and certain quality control criteria. Potentially the data required for analyses could be collected by the individual clinics via their own independent systems and then provided to the coordinating centre (e.g. as in KODAP), or the coordinating centre could make a centralized electronic data capture service available to participating clinics for the purpose of outcome monitoring. This model could provide a means to overcome one challenge for implementation and dissemination of data-intensive treatment personalization processes, that patients seen within small clinical practices may not have the same opportunity to benefit from personalization as those seen within larger treatment centers. For example, via such a research network an approach such as comparing treatment trajectories to a ‘nearest neighbour’ (Lutz et al., 2019) could be expanded across all sites, and via inclusion of local clinic characteristics (including e.g. indices of relative prosperity of their location) even patients being seen in very small clinics could benefit from a very high level of personalized evaluation informed by ‘big data’. As with the previous model discussed, randomization at the individual patient level allows individual clinics to enter and leave such a trial in a non-random way without causing problems, and it also facilitates closer examination of the extent to which the personalization method provides benefits across different sites.

### A Longer-Term Perspective

If we take a long-term view of APTs such as the leapfrog design embedded in routine practice, and imagine this approach extended over time as a perpetual process, there are of course other considerations that come into play. For any particular clinical outcome, there will likely be ceiling effects, and it may be that at certain points the set of analysis parameters will need to be adjusted (to detect smaller effect sizes), or the target outcome will need to be switched (or combined into a composite of some kind). In fact, from a long-term perspective it might be more feasible *not* to focus on the primary clinical outcome as the target for success. Rather, the focus could instead be on targeting outcomes or treatment components where there is greatest room for improvement, while maintaining non-inferiority (as a minimum requirement) on the primary clinical outcome. For example, with an internet-delivered CBT intervention it may be that for a certain period the leapfrog trial focuses on personalization methods specifically targeted at increased engagement (however this is operationalized) in one specific module of the treatment. That is, for a period the primary outcome for which the BF is calculated would be a very specific index of engagement in this one module (for which even medium effect size improvements may be achievable, requiring much less time and participant resources). Tweaked versions of the intervention reaching BF_success_ for this outcome (while meeting a pre-set criterion for non-inferiority on the clinical primary outcome) would then become the new comparator. Once it seems unlikely that further improvement on this specific aspect can be achieved, the focus for making tweaks (and the outcome measure used for calculating the BFs) could be moved to another specific component or aspect of the intervention. Over time, the benefits of these very small tweaks may then accumulate and contribute to small increases in outcomes on the primary clinical outcome that may be undetectable in individual trials but provide significant improvements at a population level.

Another longer-term consideration is that over time, ‘incorrect’ decisions will certainly be made; that is, there will be treatment arms that hit the criterion for success and become the new comparator arm without in fact being meaningfully superior.^6^ To some extent, this consideration is nothing to do with or specific to the leapfrog design or APTs themselves, but simply an inevitable consequence of testing many treatments over time.^7^ An advantage with seeing the leapfrog design as a continuous process is that making incorrect decisions has less cost, as these will be due to ‘unlucky’ sampling for a given time period; over time the arm that has won incorrectly will be overtaken by another. Similarly, if an arm is incorrectly rejected, it probably did not reflect the only chance to improve upon what was then the standard treatment. Further, if sufficient evidence arises that perhaps some new variant of this incorrectly-rejected arm could in fact be useful, it can be tried again. However, if one treatment arm is so much better than the other arms that missing it would be seen as a huge loss, a leapfrog trial designed to detect small effect sizes will have very high sensitivity to detect it (e.g. Table 1 shows that for effect sizes above the effect size that the trial is powered to find, e.g. in this case of *d* = 0.3 or above, the probability of not detecting superiority is very low). From a longer time perspective, the results of individual comparisons are less important than the general movement in the direction of improvement. As demonstrated in the simulations accompanying the protocol for Blackwell et al. (2023), the researcher can simulate a longer trial process with multiple arms over a range of likely effect size distributions and calculate the probability that at any one point in time (e.g. after 5 arms, after 10 arms etc.) the treatment currently used as control is in fact superior to the original starting point, and by how much, and this is probably a better guide to planning than focusing on the individual comparisons themselves.

As a final consideration, the kinds of combined multi-site/multi-service treatment development and roll-out models outlined above would of course come with many logistical challenges to think through. However, if an approach is to become successful and implemented, trying to implement it across settings is not something that can be avoided; embedding this dissemination into an leapfrog design thus combines testing feasibility of implementation with evaluation of efficacy. This could help avoid research waste that can otherwise occur when substantial resources are invested in early-phase research into an approach that is ultimately doomed for feasibility or acceptability reasons (e.g., see Fig. 1 in Blackwell et al., 2019). Routine psychological therapy delivery is thus converted into a dynamic process for treatment optimization.

## Challenges and Caveats to the Application of the Leapfrog Design to Treatment Personalization

There are a number of challenges and caveats that apply to the application of the leapfrog design to treatment personalization as described in this paper, and therefore need to be taken into account in the planning process.

A first challenge is that although with a leapfrog trial the sample sizes needed may be much reduced compared to standard research pathways, there is no getting away from the need for large sample sizes if you are looking for small effects (which is always likely to be the case here). Further, although on average a leapfrog design will result in a smaller sample size, this is probabilistic: if unlucky, for any one comparison between arms the final sample size could end up being the same or even greater than the one from a standard power calculation. However, from a longer-term perspective a leapfrog trial will ultimately end up being more efficient, and the potential resource-savings become more and more substantial the smaller the effect sizes being chased.

A second factor to take into consideration is that the efficiency savings of the leapfrog design will vary considerably depending on the interplay between the rate of recruitment and time to acquire the primary outcome (see e.g., Blackwell et al., 2023). As an extreme example, if a primary outcome of long-term success is chosen, and recruitment proceeds so fast that all participants are recruited before N_min_ is reached, the design offers no additional efficiency in terms of participant numbers. Hence these factors must be taken into account when deciding whether to use such a design and how to apply it, and it might be that other flexible designs are sometimes more advantageous (e.g., group sequential designs; see Huckvale et al., 2023, for an example applied to psychological therapy). However, this consideration does not mean that only short-term success can be chosen as the primary outcome, simply that the effect of outcome measure selection on the trial’s efficiency needs to be considered. There may also be ways to model longer-term outcomes more efficiently. For example, if participants complete multiple assessments over time, then treatments can be compared in terms of rate of change (with time as a continuous variable) and participant data can be added as it accumulates (while participants are still in treatment). Further, more complex Bayesian models in which measurements at intermediate time points are included as predictors of the final longer-term outcome can be used (Angus et al., 2019; Wason et al., 2015). Finally, if a new treatment is superior to the comparator only early on in treatment but equivalent in the longer-term, this still reflects a potentially valuable improvement; given the choice most people would probably choose a faster recovery even if their final endpoint will be the same.

As a third caveat, when considering trials in which termination of data collection is determined by interim analyses, for example via sequential Bayesian analyses, it is always worth remembering that the resulting effect size estimates can be biased (Schönbrodt et al., 2017). This means that the point estimates must be treated with some caution and uncertainties about the precision and likely values of effect sizes taken into account if these are to be used in decision-making.

A fourth caveat is that the timing of the implementation of additional treatments in a leapfrog trial is not completely predictable. This adds an additional complicating factor to some of the challenges common across all types of therapy trials, such as training, supervision, and fidelity monitoring, and blinding of investigators to results. However, the speed at which any changes must be made will probably match the complexity of the changes needed to some extent. For example, if the treatments being tested are face-to-face therapies, and training in a new method or technique is needed before implementing a new arm, this may require more time than e.g. simply switching in a new version of an automated computerized intervention. However, the through-flow of patients in a face-to-face therapy study will also probably be slower than that through a computerized intervention study. Further, even if there is a delay between one treatment dropping out and another being introduced, the consequences will probably not be too severe. For example, while therapists are being trained other treatment arms could still proceed as normal; if there was only one other arm, recruitment could be paused temporarily until preparations have been completed. However, all these practical aspects should ideally be planned ahead before starting the trial and reviewed in an ongoing process.

As a fifth caveat, personalization approaches tend to end up adding further complexity into the clinical decision-making process and provision of services. Planning and carrying out the analyses required for testing personalization approaches may require bringing in new staff with sufficient statistical expertise, although if a clinic is part of a larger consortium this expertise can of course be shared and centralized within the coordinating centre. Additionally, there is a danger that over time the complexity involved in delivering personalized treatments simply accumulates further and further as one approach builds upon another. Further, given that the increments in effectiveness expected are relatively small, one could argue that personalization is a relatively inefficient way to improve mental health outcomes, compared to, for example, simply trying to increase the proportion of people who receive an evidence-based treatment of some kind (currently very low, e.g. Singla et al., 2023), including via dissemination of low-cost scalable interventions (e.g., Loades & Schleider, 2023). However, wherever therapies are being delivered and received we should be trying to optimize this delivery to make the best use of the limited resources available, and personalization offers a potentially valuable way to achieve this aim. The increases in efficiency brought about by successful personalization may be particularly relevant for higher-cost therapies, such as those delivered in-person and face-to-face, but of course can also be applied to low-cost scalable digital interventions. From these perspectives it would be useful to collect the data necessary to inform cost-effectiveness analyses (e.g., Delgadillo et al., 2021), and at some point perhaps even consider stripping out additional ‘features’ that have accumulated over time to see whether some can now be removed, and the treatment simplified, without losing treatment efficacy. For example, as noted by Blackwell et al. (2019), it would also be possible to conduct a leapfrog trial based on non-inferiority rather than superiority, by calculation of the appropriate Bayes factors (see e.g., van Ravenzwaaij et al., 2019), providing a way to test out stripping back interventions or even formal dismantling studies within routine care. As mentioned previously, we cannot assume the benefits of one particular personalization approach will be stable over time; a personalization method that at some point provided great benefits in efficacy may later cease to have any impact, for example if the impact of factors external to therapy (e.g. changes in welfare/social security or health service provision, national or global instability) on mental health outcomes overwhelm the impact of small changes in therapy procedures.

Finally, although the focus of this paper is the leapfrog design, before leaving the discussion of challenges and caveats it is worth noting some more general challenges in relation to testing and implementing personalization approaches (for recent more detailed discussion and elaboration see e.g., Deisenhofer et al., 2024; Douglas et al., 2023). In general, evaluation of treatment outcomes and implementation of personalization approaches based on patient characteristics will require extensive data collection that may be additional to that routinely collected in many treatment settings. Further, comparison of different treatment approaches will often require some standardization (and fidelity monitoring) of therapy procedures. In many contexts, introduction of such features may be met with resistance by clinicians, especially in circumstances where there is already skepticism with regard to evidence based approaches in general (see e.g., Lilienfeld et al., 2013). This additional measurement and monitoring may mean additional time burden, and the introduction of formalized treatment personalization may be seen as undermining clinicians’ expertise. It may therefore be that in some treatment settings a huge amount of work is needed to achieve the basic foundations for treatment evaluation before any formal research work can be undertaken. Frameworks provided by implementation science (e.g., Aarons et al., 2011; Damschroder et al., 2009) may be useful in planning such work. It would also be important to try to involve clinicians as far as possible in planning and implementing any changes, as well as taking on board perspectives from patients in order to optimize how the changes are introduced and explained (see e.g., Barkham et al., 2023; Douglas et al., 2023; Gómez-Penedo et al., 2023; Woodard et al., 2023, for some recent discussion of these issues in relation to routine outcome monitoring, as one example). These challenges are not specific to taking a leapfrog approach, but rather apply to the implementation and testing of treatment personalization approaches in general. However, as noted earlier, it is possible that introducing and evaluating such changes within the context of a leapfrog trial may carry some advantages. For example, if a clinic is aiming to join a consortium, the flexibility in when and how they can join enabled by the sequential analyses reduces the time pressure that often comes with signing up for a trial and means the clinic can make the changes needed at their own pace. Further, the trial reflects a continuous process of trying to find ways to improve outcomes rather than top-down imposition of a ‘better’ way of providing therapy. In fact, feedback from clinicians and patients could be used to improve upon the treatment personalization approach and inform the design of new treatment arms (and may be necessary to improve usability and acceptability). Thus the participation in the leapfrog trial would ideally not simply be a one-way imposition on clinicians or patients, but involve them in actively shaping the approach taken. Especially in cases where the clinic is part of a larger consortium, this could include creating spaces (e.g. one-day conferences or workshops) to bring together wider networks of clinicians and patients for discussion and exchange of ideas. As a final point, even if the process of starting or becoming involved in a leapfrog trial takes a long time, the preparatory steps towards this aim are likely to bring benefits in themselves, for example via incorporation of more systematic outcome measurement, or better orientation towards evidence-based approaches; just because the final destination is a long way off this should not discourage us from attempting to move in the right direction.

## Conclusions

While personalization of psychological treatments provides an especially promising way forwards, it also presents particular challenges for testing, development, and implementation. Incorporation of adaptive platform designs into routine practice, with the ‘leapfrog’ being a simple exemplar, provides a way to streamline this process and bridge links between innovation and implementation to improve outcomes. Although consideration of the leapfrog design and its application to treatment personalization raises many challenges and complexities, most of these are not specific to the design itself. Rather, these are inevitable consequences of taking a longer-term view of treatment development, as opposed to thinking about just one trial at a time. However, the leapfrog design allows longer-term planning to be combined with ongoing flexibility and responsivity to changes in service demands and treatment priorities over time. Its integration into routine practice therefore offers the opportunity to transform ongoing treatment delivery into a continuous process of treatment development and optimization, facilitating not only research progress but improved real-world treatment outcomes.

## Data Availability

Simulation scripts for this article can be found at https://osf.io/fe4ck/.

## Footnotes

1 The term ‘routine practice’ is here used broadly to refer to any setting in which people presenting for treatment would routinely receive psychological interventions, i.e. not only limited to research clinics, but including public and private clinics and psychological therapies services. In this way, ‘routine practice’ refers to the real-world settings in which we would ultimately want personalization to be implemented in order to have a meaningful effect on treatment outcomes.

2 To keep things simple for illustrative purposes we assume no missing data. Of course, for a real trial this would not be a realistic assumption, and as shown by Blackwell et al. (2023) you can simulate across different levels of missing data for a leapfrog trial. However, it is hoped that focusing on those factors specific to the leapfrog design rather than those that are ubiquitous across all clinical studies (such as missing data) helps maintain the clarity of the paper.

3 Note that by the ‘standard’ therapy in this example we mean the treatment as currently provided in the therapy service. This could be standardized, manualized, therapies informed by structured assessments, eclectic mix-and-match approaches informed by the assessing clinician’s clinical judgement, or anything else; for the purpose of this example we simply use this term to refer to what is ‘standard’ at that service at the start of the trial, i.e. the current state of affairs that you wish to improve upon.

4 See https://osf.io/fe4ck/ for R scripts used to create this table. Note that here we have also specified the ‘stepsize’ parameter in BFDA at 10, meaning the analyses are repeated every 10 participants per arm. This is in part to reduce the time needed for the simulations, but may also be realistic in such a trial; when the sample sizes are likely to be in the order of hundreds of participants, there is less to gain from repeating the analyses every single time a new data point comes in compared to every 5 or 10 participants.

5 In fact, this principle can also be applied to other trial designs that may be used to investigate personalization (see e.g., Sauer-Zavala et al., 2022; Watkins et al., 2023, for other options); by implementing the primary analysis within a Bayesian framework and applying it sequentially, the conclusions from such trials could potentially be reached more efficiently.

6 Note that when using directional BFs it is highly unlikely that an arm would hit the success criterion if in fact meaningfully *inferior* to the comparator arm, so the risk of backwards progress is so small as to be negligible.

7 Theoretically for one given trial with a known number of arms it would be fairly simple to control the family-wide error rate via selection of the analysis parameters, and even with an unknown number of arms (e.g. a perpetual trial) it would be possible to implement a dynamic false-discovery control procedure (as for frequentist approaches, e.g. Javanmard & Montanari, 2018). However, if the leapfrog design is seen as an alternative to what would normally be a sequence of independent trials happening one after the other, it is questionable whether this really makes sense. For example, if a researcher conducts 10 RCTs over the course of their career they do not adjust the p-value thresholds from one trial to the next to reflect that they have 9 trials to go, or that they have now completed 7 trials with a certain distribution of p values. Similarly, a researcher planning their first trial does not decide to use a adjusted p-value threshold because they plan to carry out several more over the course of their career. Rather, each trial is taken as it comes, and hence it makes sense to do the same with the leapfrog design: each pairwise comparison has a known, fixed, error rate, and the consequences of any false-positive errors made will be corrected for over time.
